# Cognitive performance and behavior across idiopathic/genetic epilepsies in children and adolescents

**DOI:** 10.1038/s41598-020-78218-0

**Published:** 2020-12-09

**Authors:** Frederik Jan Moorhouse, Sonia Cornell, Lucia Gerstl, Moritz Tacke, Timo Roser, Florian Heinen, Michaela Bonfert, Celina von Stülpnagel, Matias Wagner, Ingo Borggraefe

**Affiliations:** 1 Division of Pediatric Neurology, Developmental Medicine and Social Pediatrics, Department of Pediatrics, Dr. von Hauner Children’s Hospital, Ludwig-Maximilians-University, Lindwurmstraße 4, 80337 Munich, Germany; 2 Paracelsus Medical University, Salzburg, Austria; 3 Institute of Human Genetics, Klinikum Rechts Der Isar, Technical University of Munich, Munich, Germany; 4 Institute for Neurogenomics, Helmholtz Zentrum München, Neuherberg, Germany; 5 Comprehensive Epilepsy Center (Pediatric Section), Ludwig-Maximilians-University, Munich, Germany

**Keywords:** Intelligence, Epilepsy, Genetics of the nervous system, Human behaviour

## Abstract

We investigated the cognitive and behavioral profile of three distinct groups of epilepsies with a genetic background for intergroup differences: (1) idiopathic/genetic generalized epilepsies (IGE/GGE group); (2) idiopathic focal epilepsies (IFE group); and (3) epilepsies with proven or strongly suggested monogenic or structural/numeric chromosomal etiology (genetic epilepsies, GE group). Cognitive (total IQ and subcategories) and behavioral parameters (CBCL) were assessed at the tertiary epilepsy center of the University of Munich (Germany). We used ANOVA with post-hoc Bonferroni-correction to explore significant mean differences and Fisher’s exact test for significant proportional differences of intelligence impairment and behavioral problems. 126 (56 IGE/GGE, 26 IFE, 44 GE) patients were available. Total IQ was 89.0 ± 15.9 (95% CI 84.5–93.4) for IGE/GGE, 94.8 ± 18.1 (95% CI 87.3–102.3) for IFE and 76.4 ± 22.4 (95% CI 67.6–85.3) for GE (*p* = 0.001). The same trend was significant for all but one IQ subcategory. The rate of patients with an intelligence impairment (total IQ < 70) was higher for GE (40%) than for IGE/GGE (14%) and for IFE (7%) patients (*p* = 0.033). There were no significant differences between groups for behavior scores and behavioral problems. This study shows that the current ILAE classification of epilepsies with genetic etiology creates a heterogeneous group of patients with respect to cognitive performance but not behavior. These findings may help in further delineating epilepsies as regards cognitive performance, notwithstanding their closely related etiological classification.

## Introduction

Epilepsy is one of the most prevalent neurological diseases in childhood and adolescence. Cognitive impairments and behavioral issues are common among children with epilepsy and occur more often than in the general population^1^. Additionally, epilepsy patients often feel more socially disadvantaged than patients with other chronic diseases^2^. Both cognitive impairment and behavioral issues affect quality of life of patients as well as of parents and caregivers^3^. Cognitive impairment in epileptic children is a risk factor for less educational success. Furthermore, adult patients of childhood-onset epilepsies with cognitive problems have a higher rate of unemployment than childhood-onset epilepsy patients without cognitive problems^4^. In children with epilepsy, the presence of cognitive or behavioral comorbidities has been shown to be more relevant for quality of life than seizure frequency or seizure freedom^5^.

The increasing knowledge about genetically-caused epilepsies has changed the perspective on classifying and treating epilepsies of genetic origin. On the one hand, there are clearly defined genetic epilepsies (GEs) with proven or strongly suggested monogenic causes or structural and numeric chromosomal aberrations. On the other hand, there is a strong suspicion that idiopathic epilepsies have complex underlying polygenic and epigenetic mechanisms and monogenic causes are only seen in a minority of patients^6^. These mechanisms are more prevalent in idiopathic generalized epilepsies (IGEs) than in idiopathic focal epilepsies (IFEs), which led to the recent change in ILAE classification, suggesting that the term “Genetic Generalized Epilepsies” (GGE) replaces the term IGE^7^. However, intractable seizures and cognitive impairments are very frequent in GEs which appears not to be true for the majority of patients with idiopathic epilepsies^8^. Thus, lumping together rather self-limiting or (in the majority of cases) convenient to treat epilepsies such as IGE/GGE and IFE, and the more challenging group of GE may confuse both patients and parents and may present challenges when counselling patients.

The aim of the present study was to investigate cognitive and behavioral patterns of three distinct groups of epilepsy with a genetic background for intergroup differences: (1) idiopathic generalized epilepsies/generalized genetic epilepsies (IGE/GGE group); (2) idiopathic focal epilepsies (IFE group); and (3) epilepsies with proven or strongly monogenic or structural chromosomal etiology (GE group).

## Methods

### Data collection

Patient data were collected by searching the database of a tertiary pediatric epilepsy center of the University of Munich (Germany) for patients who fulfilled the following inclusion criteria: age between 6 and 17 years at testing, epilepsy diagnosis and previously undergone neuropsychological testing. Exclusion criteria were: epilepsies not classified as IGE/GGE, IFE or GE, and neuropsychological test results without any Intelligence Quotient (IQ) and Child Behavior Checklist (CBCL) scores. We also evaluated sub-categories of IQ and CBCL. Thus, an available result in a single (sub-)category sufficed for inclusion. Therefore, not all variables have scores for every participant. Epilepsy classification was reviewed and reevaluated by three board-certified epileptologists (IB, MT, TR).

The following data were transferred into an excel-based spreadsheet: sex, age at inclusion, epilepsy classification, age at epilepsy diagnosis, epilepsy duration, seizure frequency and number of Antiepileptic Drugs (AEDs) along with IQ test values and T-value-normalized CBCL scores. Names were replaced by randomized case numbers.

### Cognitive evaluation

IQ tests were performed in German using the Wechsler Intelligence Scale. The Wechsler Intelligence Scale, fourth and fifth editions (WISC-IV, WISC-V), were used for children between six and 16 years^9,10^. The Wechsler Preschool and Primary Intelligence test, third and fourth editions (WPPSI-III, WPPSI-IV), were used for children aged six and seven years who did not yet attend school at the time of evaluation^11,12^. The “Wechsler Intelligenztest für Erwachsene” (WIE), which is the German adaptation of the Wechsler Adult Intelligence Scale, third edition (WAIS-III), and the Wechsler Adult Intelligence Scale, fourth edition (WAIS-IV), were used for adolescents aged 17. It was also used for adolescents aged 16 when questions of higher education and training arose^13,14^. The following IQ subcategories were evaluated beside the total IQ as Index-values: verbal IQ (verbal-index; overall verbal intellectual abilities such as language comprehension, acquired knowledge and verbal reasoning as Index-value), non-verbal IQ (nonverbal-index; organization, thinking skills, conceptual reasoning and problem-solving abilities relating to visual information etc.), working memory IQ (working memory-index) and processing speed IQ (speed-index).

### Behavioral evaluation

The behavior of patients was assessed using the German versions of the CBCL/4-18 and CBCL/6-18R parent questionnaires^15,16^. Test results were noted as T-values (average: 50; standard deviation (SD): 10) for easier and better comparison of patients’ results. In the case of CBCL, results are not interpreted by distance in SDs to average but by a cut-off: T-Values > 65 are considered to be clinically relevant problematic behavior. The three main categories of the CBCL were noted in the spreadsheet: internalizing, externalizing and total behavior.

### Statistical analysis

Excel was used as a basis for the spreadsheet and for the figures. Statistical analysis was performed using SPSS v.26. The Kolmogorow–Smirnow-test was used to test the total cohort results for normal distribution. Analysis of Variance (ANOVA) with post-hoc Bonferroni correction was used to test the groups for significant intergroup differences in IQ scores and CBCL T-Values. Cross tables were used to compare the number of at least mildly intelligence-impaired children and the number of children with behavioral problems across epilepsy groups. Fisher’s exact test was used to determine the significance of intergroup differences. ANOVA was again used to test sex, age at inclusion, age of epilepsy diagnosis and epilepsy duration for intergroup differences. The Kruskall–Wallis-test was used to test whether the number of AEDs or seizure frequency exhibited significant intergroup differences. For all tests, we chose α = 0.05.

### Data protection and ethics

After data for each patient had been collected and revisions on the data were completed, patients’ names were completely anonymized by a random code generated by MD5Hex. Due to the complete anonymization of data, Informed Consent was waived by, and the study approved by the local ethical board of the Medical Faculty of the University of Munich (#20-150). The study adheres to the guidelines for medical research set out by the declaration of Helsinki and its amendments.

## Results

### Demographic data

**Figure 1:**
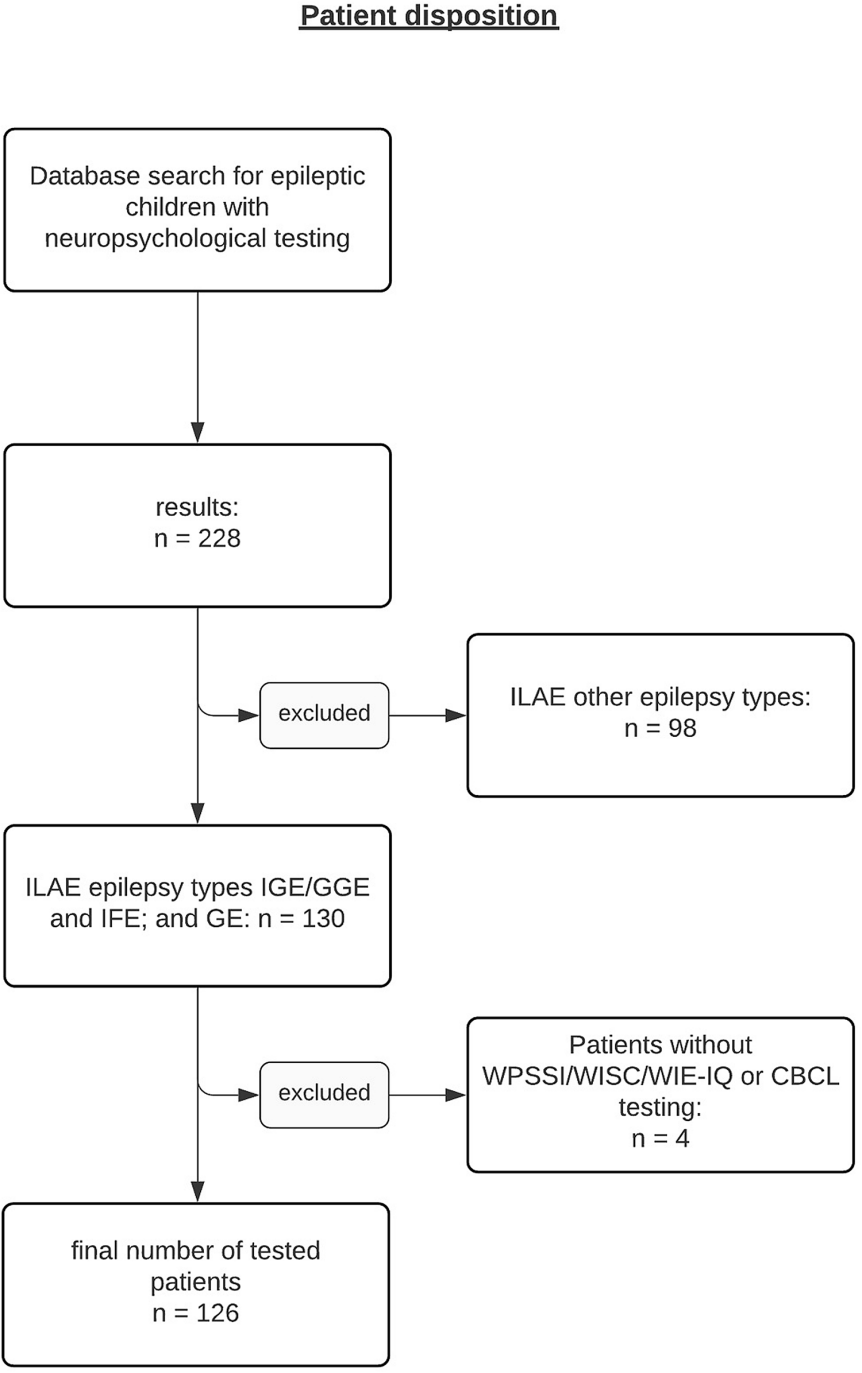
Patient disposition showing step-by-step selection process with inclusion and exclusion criteria.

228 epilepsy patients aged 6–17 who had undergone neuropsychological testing were identified. After exclusion of epilepsies that could not be classified as IGE/GGE, IFE or GE and patients without IQ or behavior testing, a remaining sample of 126 patients was available for final data analysis (Fig. 1).

A detailed description of demographic data is depicted in Table 1. The three study groups were distributed as follows: 56 (44.4%) were classified as IGE/GGE, 26 (20.6%) as IFE and 44 (35.0%) as GE. Patients with GE revealed an earlier age at diagnosis, longer epilepsy duration at the time of inclusion and higher seizure frequency and AED load compared to patients with IGE/GGE and IFE. Significant intergroup differences were found for all variables except sex.

**Table 1:** Demographic data.

	Total	IGE/GGE	IFE	GE	*p* values
Epilepsy type (n)	126	56	26	44	
**Sex (n)**					
m/f	67/69	34/22	11/15	22/22	*p* = 0.265
**Average age at inclusion in years**					
Mean (95% CI); SD	10.0 (9.3–10.6); 3.5	10.9 (9.9–11.9); 3.7	9.1 (8.1–10.1); 3.4	9.3 (8.3–10.3); 3.5	*p* = 0.022
**Average age of epilepsy diagnosis in years**					
Mean (95% CI); SD	5.8 (5.1–6.6); 4.1	7.3 (6.2–8.3); 4.1	6.0 (4.8–7.2); 2.8	4.0 (2.8–5.3); 4.0	*p* < 0.001
**Average epilepsy duration in years**					
Mean (95% CI); SD	4.2 (3.5–4.8); 3.4	3.7 (2.9–4.5); 3	3.3 (2.1–4.4); 2.9	5.3 (4.0–6.5); 4.0	*p* = 0.029
**Seizure frequency (n)**					
Not reported	7	5	0	2	*p* = 0.035
Seizure free	64	34	15	15	
1/(6 M)	22	4	7	11	
1/M	7	2	1	4	
Weekly	10	2	2	5	
Daily	17	9	1	7	
**Number of AEDs (n)**					
Not reported	1	0	0	1	*p* = 0.021
0	24	5	10	9	
1	63	36	12	14	
2	27	11	2	14	
3+	12	4	2	6	

IGE/GGE, idiopathic/genetic generalized epilepsy; IFE, idiopathic focal epilepsy; GE, genetic epilepsy; m, male; f, female; CI, confidence interval; SD, standard deviation; M, month(s); AED, antiepileptic drug.

IGE/GGE and IFE were further classified into distinct subtypes. Monogenic causes were the most common findings within the GE (n = 30) cohort besides either structural or numeric chromosomal causes (n = 9) and cases without proven mutations but with diagnosed syndromes or strong suspicions due to the clinical manifestations of the diseases (n = 5) (Table 2). The proportion of patients with available genetic testing within the IGE/GGE and IFE group was 20.5%, with the majority of these patients receiving either exome or epilepsy panel investigations (94.1%). None of the patients of the GE group was eligible for either classification as IGE/GGE or IFE.

**Table 2:** Epilepsy syndrome classification.

IGE/GGE	IFE	GE
n.f.s (n = 14) CAE (n = 19) JAE (n = 4) JME (n = 9) MAE (n = 7) ELMA (n = 1) EMA (n = 1) BME (n = 1)	BECTS (n = 24) ABPE (n = 2)	**Monogenetic causes (n = 30)** TSC (n = 7), SCN1A (n = 6), CLN3 (n = 2), RORB (n = 1), SCN9A (n = 1), FASTDK2 (n = 1), FMR1 (n = 1), NPRL3 (n = 1), MECP2 (n = 1), FOXG1 (n = 1), CDLK5 (n = 1), KCNQ2 (n = 1), NRNX1 (n = 1), SLC13A5 (n = 1), GABRB3 (n = 1), DCX (n = 1), GLUT1 (n = 1), ADSL (n = 1) **Structural or numeric chromosomal causes (n = 9)** Ringchromosome 20 (n = 1), Deletion 18q22.1q23 (n = 1), Deletion 2p24.2 (n = 1), Deletion 15q13.3 (n = 1), Paternal deletion 15q11.2-q13 (n = 1) Deletion 2p25.3p25, Duplication 6p21.1 (n = 1), Deletion 17q12, Duplication 17q21.31 (n = 1), Deletion 2q37 (n = 1), Duplication 5q35.3 (n = 1) **Others (n= 5)** clinical diagnosis of Dravet syndrome without proven mutations (n = 2), clinical diagnosis of GEFS + syndrome without proven mutations (n = 1), strong clinical suspicion of a genetic syndrome due to associated malformations but without proven genetic cause (n = 2)

IGE/GGE, idiopathic/genetic generalized epilepsy; IFE, idiopathic focal epilepsy; GE, genetic epilepsy; n.f.s., not further specified; CAE, childhood absence epilepsy; JAE, Juvenile Absence Epilepsy; JME, Juvenile Myoclonic Epilepsy; MAE, Myoclonic Absence Epilepsy; ELMA, Epilepsy with Myoclonic Absences; EMA, Eyelid Myoclonia with Absences; BME, Benign Myoclonic Epilepsy; BECTS, Benign Epilepsy with Centro-Temporal Spikes; ABPE, Atypical benign partial epilepsy; TSC, Tuberous Sclerosis Complex; GEFS+, Genetic Epilepsy with Febrile Seizures+, all others are abbreviations of genes adhering to the HUGO Gene Nomenclature Committee’s guidelines^39^.

### Cognitive performance

**Figure 2:**
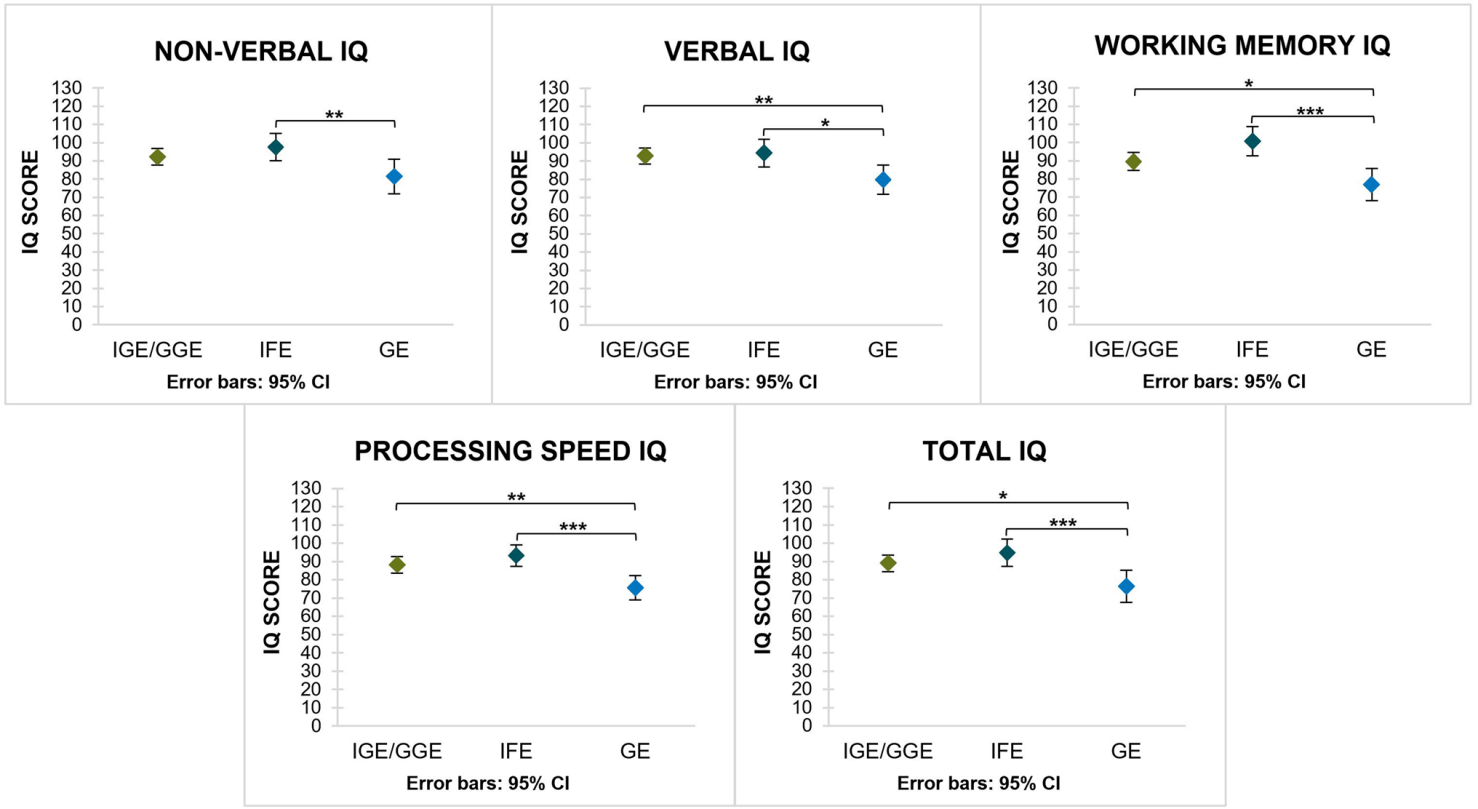
IQ means of epilepsy groups for all IQ categories; significant differences marked by bars and asterisks, non-significant differences are not shown. *p ≤ 0.05; **p ≤ 0.01; ***p ≤ 0.001.

**Figure 3:**
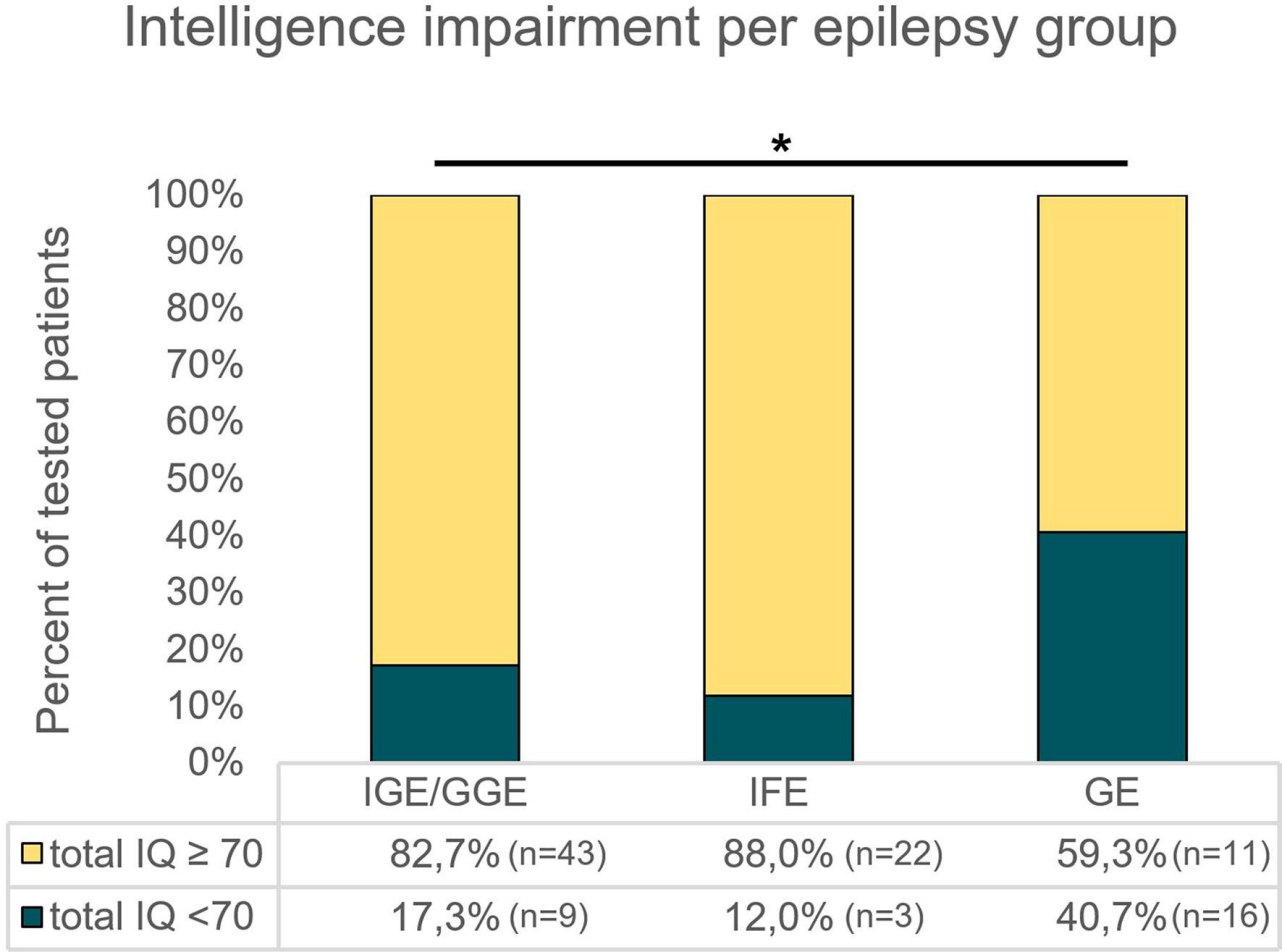
Intelligence impairment comparison in percentage of impaired children per epilepsy group with total numbers included in accompanying table. *p = 0.033.

Data for all IQ scales were normally distributed. Participation rates—noted as: subjects per parameter (non-verbal IQ, verbal IQ, working memory IQ, processing speed IQ, total IQ)/cohort size—were: (50, 52, 41, 50, 52)/56 for IGE/GGE; (25, 26, 19, 25, 25)/26 for IFE and (26, 31, 22, 28, 27)/44 for GE, respectively. Total IQ was 89.0 ± 15.9 (95% CI 84.5–93.4) for the IGE/GGE, 94.8 ± 18.1 (95% CI 87.3–102.3) for the IFE and 76.4 ± 22.4 (95% CI 67.6–85.3) for the GE group, respectively, and differed significantly between groups (*p* = 0.001) (Table 3). Significant intergroup differences were also found for all other IQ subtypes (Table 3). Post hoc analyses revealed significantly lower results for total IQ and all but one IQ subcategory (non-verbal IQ) of the GE cohort compared to both the IGE/GGE and IFE cohorts (Fig. 2). The rate of patients with an intelligence impairment (total IQ < 70) was significantly higher (*p* = 0.033) in GE patients (40%) than IGE/GGE (14%) and IFE (7%) patients (*p* = 0.033) (Fig. 3). The same applies for all IQ subcategories.

**Table 3:** Cognitive performance across groups.

	IGE/GGE			IFE			GE			*p value*
	Mean ± SD	Range (Min;Max)	95% CI	Mean ± SD	Range (Min;Max)	95% CI	Mean ± SD	Range (Min;Max)	95% CI	
Non-verbal IQ	92.2 ± 16.0	57;125	87.6–96.8	97.6 ± 18.1	65;129	90.1–105.1	81.4 ± 23.5	47;129	71.8–90.1	*p* = 0.008
Verbal IQ	92.7 ± 15.7	59;128	88.4–97.1	94.4 ± 19.0	54;126	86.7–102.1	79.8 ± 21.9	45;116	71.8–87.8	*p* = 0.003
Working Memory IQ	89.6 ± 15.5	59;117	84.7–94.5	100.8 ± 16.5	62;126	92.9–108.8	76.9 ± 19.8	48;120	68.1–85.7	*p* < 0.001
Processing speed IQ	88.2 ± 16.1	50;131	83.6–92.8	93.3 ± 14.2	60;117	87.5–99.2	75.7 ± 17.2	46;117	69.0–82.3	*p* < 0.001
Total IQ	89.0 ± 15.9	54;118	84.5–93.4	94.8 ± 18.1	59;119	87.3–102.3	76.4 ± 22.4	41;127	67.6–85.3	*p* = 0.001

Intergroup differences are considered significant at *p* ≤ 0.05.

IQ, intelligence quotient; IGE/GGE, idiopathic/genetic generalized epilepsy; IFE, idiopathic focal epilepsy; GE, genetic epilepsy; CI, confidence interval; SD, standard deviation; Min, minimum; Max, maximum.

### Behavior

Data for all CBCL scales were normally distributed. Participation rates—noted as: subjects per parameter (internalizing behavior, externalizing behavior, total behavior)/cohort size—were: (47, 46, 47)/56 for IGE/GGE; (19, 19, 19)/26 for IFE and (39, 37, 38)/44 for GE, respectively. Total CBCL score was highest within the GE group and lowest in the IFE group. The same was true for the externalizing subscale, whilst IGE/GGE were lowest on the internalizing subscale (Table 4). No significant differences were found between the scores of the groups. The means of all the T-values were > 50. The proportion of tested patients with a general behavioral problem (total scale T-value > 65) was 38% (n = 18) for the IGE/GGE group, 26% (n = 5) for the IFE group and 53% (n = 20) for the GE group, respectively. The same trend was true on the internalizing and externalizing scale. No significant differences were found in the proportion of problems.

**Table 4:** Behavior across groups.

	IGE/GGE			IFE			GE			*p value*
	Mean ± SD	Range (Min;Max)	95% CI	Mean ± SD	Range (Min;Max)	95% CI	Mean ± SD	Range (Min;Max)	95% CI	
Internalising CBCL scores	59.3 ± 9.5	40;80	56.5–62.1	59.4 ± 10.1	41;77	57.5–63.5	60.6 ± 9.3	38;76	54.6–64.3	*p* = 0.844
Externalising CBCL scores	56.4 ± 11.4	37;87	53.0–59.8	54.3 ± 9.1	40;69	49.9–58.7	59.1 ± 9.2	36;80	56.0–62.2	*p* = 0.231
Total CBCL scores	60.6 ± 11.1	38;80	57.3–63.8	57.4 ± 9.7	38;71.5	52.7–62.1	63.4 ± 10.0	42;83.5	60.2–66.7	*p* = 0.115

Intergroup differences are considered significant at *p* ≤ 0.05.

CBCL, Child Behavior Checklist; IGE/GGE, idiopathic/genetic generalized epilepsy; IFE, idiopathic focal epilepsy; GE, genetic epilepsy; CI, confidence interval; SD, standard deviation; Min, minimum; Max, maximum.

## Discussion

### Demographic data

Epilepsy manifestation was earliest among GE patients. This is in line with previous studies, though means of our presented onsets/diagnoses were higher than of these studies^8,17,18^. This is most likely due to a selection bias for our cohort: younger age at onset has shown to be detrimental to disease outcome, leading to refractory epilepsy and cognitive disorders which might affect results of our GE cohort^17^. Furthermore, patients with even earlier seizure onset compared to our cohort are more likely to be cognitively more affected and might not be able to undergo a conventional neuropsychological evaluation. GE patients had a higher seizure frequency on average. Consequently, polytherapy of AEDs was more prominent compared to IGE/GGE or IFE patients, which is in line with previous reports establishing the difficult to treat nature of seizures in genetic epilepsies. As both seizure frequency and antiepileptic polytherapy affect cognition^19^, we suggest that these factors contribute to low cognitive performance in the GE cohort of this study.

In summary, significant intergroup differences present in our results can mostly be explained by previously found effects and outcomes of GE. Consequently, it is possible to conclude that our results are predominantly explained by the type of epilepsy.

### Cognitive performance

The presented data show that IQ mean was significantly lower for the GE (76.4 ± 22.4) cohort in comparison to the IGE/GGE (89.0 ± 15.9) and IFE (94.8 ± 18.1) cohorts. The latter two did not differ significantly. The risk of total intelligence impairment (IQ < 70) in GE patients was 2 times higher than in IGE/GGE patients and 3.5 times higher than in IFE patients. Findings were similar for all IQ subcategories.

The IFE cohort had the highest means and average intelligence results. The results were similar to other studies^20,21^ but were lower than those which exclude cognitive impaired patients^22,23^. 12.0% of our patients had an intelligence impairment which is similar to the rate in children with epilepsy in general^24,25^. Despite this, many remaining patients scored average or above average results which countered the influence of impairment.

IGE/GGE patients’ means lie at the lower end of the average intelligence range (IQ 85–115) and are lower than IFE results (though without statistical significance), indicating a higher burden on cognition. This aligns with other studies but is again lower than studies which exclude impaired patients^22,26^. The impairment ratio in our IGE/GGE cohort is 17% which is again similar to children with epilepsy in general^24,25^.

GE IQ means were in the lower than average range (IQ 70–85), which together with the higher proportion of impairment suggests that GE patients generally have a higher risk of cognitive problems. As mentioned above, earlier age of onset, a higher burden of seizure frequency and total amount of AED intake are likely to contribute to cognitive impairment. Furthermore, the neurobiology of monogenic or structural/numeric chromosomal changes is sufficient to cause significant cognitive impairment itself. This is shown by, for example, recent studies on patients with Dravet Syndrome revealing that epilepsy specific parameters are not capable of explaining cognitive decline alone^27,28^. Furthermore, animal models of *SCN1A*^*(−/*+*)*^ knockout mice reveal cognitive issues despite low seizure burden and researchers have suggested that this is due to high prevalent forebrain expression of the *SCN1A* encoded sodium channel Na_v_1.1^29^. Thus, it has been suggested to call the majority of monogenic or structural/numeric chromosomal epilepsies “Developmental Brain Disorders” with the comorbidity of epilepsy^30^. Furthermore, the ILAE coined the definition of DEE (Developmental and Epileptic Encephalopathy) which describes a portion of our GE patients^7^. Our IQ results align with a study which tested epilepsies in general, which were difficult to treat, like most of our GEs^19^. In our study, the rate of impairment is lower than in studies focusing on syndromes or mutations related to DEEs^31,32^. A possible reason for this may be the heterogeneity of our GE cohort. Consequently, DEE patients’ severe issues often make them ineligible for conventional testing and thus the tested group mainly comprises “less”, but still, impacted GE patients. As a result, our means may be higher than would be the case if the ineligible patients could be tested. This could downplay the difference between groups. The combination of how the GE means are composed and the ratio of intelligence impaired children (Fig. 3) highlights that not all GE patients have impacted cognition but that the risk of a serious effect on cognition is significantly higher in children with GE. This is even true when excluding the few tested DEEs, which are impacted in cognition by definition^7^. Despite low cognitive performance within the GE cohort, the data show that some GE patients might show normal or above average performance.

The findings that patients with genetically proven monogenetic epilepsies reveal low cognitive performance and that patients with IGE/GGE or IFE perform better are not new. Nevertheless, to our knowledge, this has not been investigated in a comparative study so far. Thus, the present data should be interpreted as confirmatory investigation of relationships suspected before from non-comparative studies with a considerable number of subjects.

### Behavior

Total behavior as measured by CBCL T-value means did not differ significantly between cohorts. The proportion of patients with behavioral problems was highest in the GE cohort (53%, compared to 38% in the IGE/GGE and 26% in the IFE cohort), though differences between cohorts did not reach statistical significance. GE patients’ scores and ratios were, as a trend, highest on all scales.

A reason for a consistent occurrence of behavioral disturbances across the three investigated cohorts might again be a selection bias before testing. If not warranted otherwise, testing occurred in children with previously observed problematic behavior. As this would affect all three epilepsy groups, a change from a trend to significant results seems unlikely.

Our CBCL results for IFE are similar to or slightly above previous studies on IFE^23,33^. IGE CBCL results also lie slightly above previous studies^34,35^. Both IFE and IGE/GGE cohorts have more behavioral problems than reported in other studies^36^. As in cognition, behavioral problems may be more frequent than reported in other papers due to testing in a tertiary epilepsy center. Additionally, the ratio of GE patients with a behavioral problem is similar to findings of a study on the ratio of behavioral issues in Dravet Syndrome patients^37^. In contrast to cognition, parental assessment of behavior of children with severe GE cases is achievable. The reason why our findings on behavioral problems share more similarities with findings for DEE patients than our findings for cognition, may be that several of these patients could not or did not complete, or undergo IQ testing.

### Limitations

Some limitations have to be acknowledged. Firstly, sampling patient data from a tertiary epilepsy center is prone to selection bias. Acquisition of patient data at a tertiary epilepsy center may also have effects on the sample as a whole. Our sample size is comparatively small at 126 participants and it is a heterogeneous sample with a larger cohort of IGE/GEE and GE patients and a comparatively smaller cohort of IFE patients (see Table 1). Despite these biases potentially resulting in overly significant results, cognitive differences between the ”idiopathic” and GE cohorts may be even more pronounced within the “real world” population for reasons discussed above. Secondly, most IGE/GGE and IFE patients were not genetically tested. Thus, we cannot exclude that patients with monogenic or structural/numeric chromosomal causes are among these cohorts. However, a recent study by the EPI25 collective has shown that genetic variations occur in up to 17.3% of IGE/GGEs and 12.2% of IFEs^38^. Thirdly, the ratio of tested patients was lowest in the GE cohort. This aspect may again reflect that GE patients are more often severely affected and consequently not eligible for neuropsychological evaluation. Fourthly, the present data reveal that patients with genetically proven or strongly suggested epilepsies perform cognitively worse that patients with IGE/GEE or IFE. However, the present study design does not allow the conclusion that this relationship is solely dependent on the neurobiological consequences of the genetic defect. As stated in the discussion, other variables such as onset of epilepsy, seizure frequency and numbers of AEDs may contribute to these findings. Finally, our evaluation of behavioral aspects is limited due to one-off testing of behavior and only using the CBCL questionnaire as an assessment tool. Whilst it gives a good overview over the patients’ behavior, personal results can be influenced by the child’s behavior in the run up to the parents filling in the questionnaire. Furthermore, a detailed analysis of problems in specific areas of behavior using more behavioral testing tools, which could provide a more nuanced outlook on the patients’ behavior, was not made.

## Conclusion

The findings of the present study may help in further delineating epilepsies as regards cognitive performance notwithstanding their closely related etiological classification. The etiological term “genetic” is associated with strongly varying effects and outcomes for patients. Therefore, maintaining a difference between genetic epilepsies, with a recognized monogenic or structural/numeric chromosomal etiology, and idiopathic epilepsies, more likely due to a polygenic background, may help counselling patients and their caregivers with respect to different cognitive outcomes.

## Data Availability

The datasets generated during and/or analyzed during the current study are available from the corresponding author on reasonable request.
